# Inhibition by 4-(4-Bromo-2-oxo-3*H*-benzimidazol-1-yl)-*N*-(4-iodophenyl)piperidine-1-carboxamide (TH5487) of the Activity of Human 8-Oxoguanine DNA Glycosylase-1 (OGG1) for the Excision of 2,6-Diamino-4-hydroxy-5-formamidopyrimidine, 4,6-Diamino-5-formamidopyrimidine, and 8-Oxoguanine from Oxidatively Damaged DNA

**DOI:** 10.1021/acs.biochem.4c00419

**Published:** 2025-04-03

**Authors:** Pawel Jaruga, Melis Kant, Michael M. Luzadder, R. Stephen Lloyd, Istvan Boldogh, Miral Dizdaroglu

**Affiliations:** † Biomolecular Measurement Division, National Institute of Standards and Technology, Gaithersburg, Maryland 20899, United States; ‡ Oregon Institute of Occupational Health Sciences, Oregon Health & Science University, Portland, Oregon 97239, United States; § Department of Molecular and Medical Genetics, Oregon Health & Science University, Portland, Oregon 97239, United States; ∥ Department of Microbiology and Immunology, University of Texas Medical Branch at Galveston, Galveston, Texas 77555, United States

## Abstract

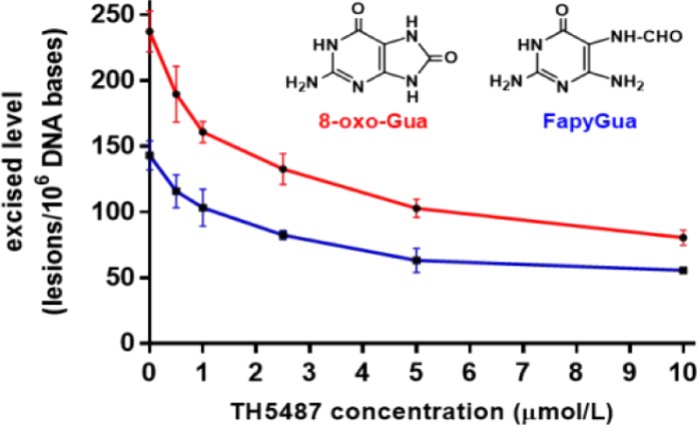

DNA glycosylases of the base excision repair pathway have become clinically validated drug targets for the treatment of several diseases. Human OGG1 (hOGG1) is specific for the removal of the highly mutagenic 8-oxoguanine (8-oxo-Gua) and 2,6-diamino-4-hydroxy-5-formamidopyrimidine (FapyGua) from damaged DNA. To develop clinically approved drugs, various small-molecule inhibitors of hOGG1 have been developed to inhibit its glycosylase and lyase activities, with 4-(4-bromo-2-oxo-3H-benzimidazol-1-yl)-*N*-(4-iodophenyl)piperidine-1-carboxamide (TH5487) shown to be a potent inhibitor. The inhibition of hOGG1 by TH5487 has been shown to suppress cancer cell growth, pulmonary inflammation, and lung fibrosis and sensitize cancer cells to ionizing radiation, confirming hOGG1 as a target for pharmaceutical intervention. While the assays that identified TH5487 utilized an oligodeoxynucleotide with the target substrate being 8-hydroxyadenine mispaired with cytosine, measurements of TH5487-mediated inhibition of the release of 8-oxo-Gua and FapyGua have not been reported. In the present work, we investigated the inhibition of hOGG1 by TH5487 using genomic DNA with multiple lesions and gas chromatography-tandem mass spectrometry with isotope dilution to measure inhibition of hOGG1-catalyzed DNA base lesion removal from DNA. An oligodeoxynucleotide containing 8-oxo-Gua was also used to measure the half-maximal inhibitory concentration (IC_50_), which is 0.800 μmol/L ± 0.061 μmol/L. We show that TH5487 efficiently inhibits the excision of both 8-oxo-Gua and FapyGua, and a minor substrate 4,6-diamino-5-formamidopyrimidine (FapyAde) from DNA with the IC_50_ values of 1.6 μmol/L, 3.1 μmol/L, and 3.1 μmol/L, respectively. The results suggest that the approach used in the present work may be applied for future studies of hOGG1 inhibition by TH5487 on cellular and animal disease models.

## Introduction

**Figure 1:**
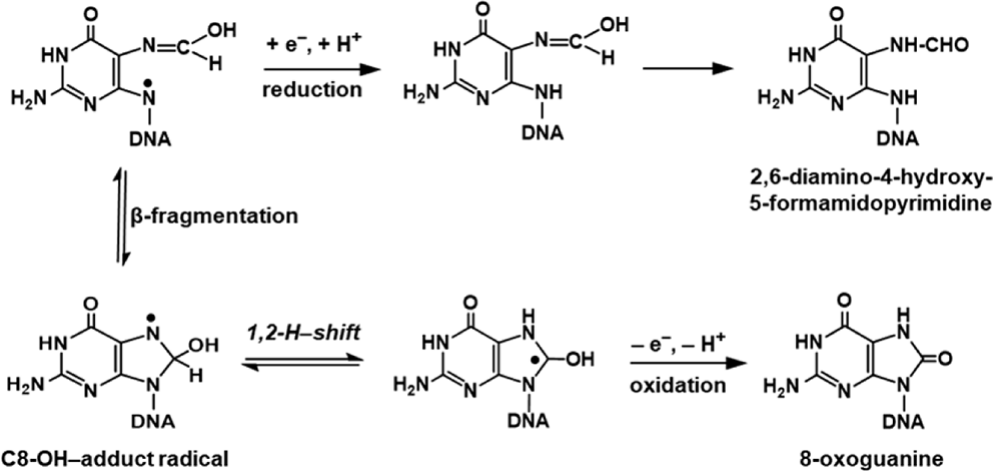
Mechanism of formation of 8-oxo-Gua and FapyGua from the C8-OH–adduct radical of guanine, which is generated by the addition of hydroxyl radical to the C8 position of guanine (adapted with permission from ref (3)). Reprinted (adapted) with permission from Dizdaroglu, M. and Jaruga, P. (2012). Mechanisms of free radical-induced damage to DNA. *Free Radical Research*, 46(4), 382–419. 10.3109/10715762.2011.653969. Copyright 2012 Taylor and Francis Group, https://www.tandfonline.com).

The maintenance of an organism’s overall genetic integrity is key for cellular physiological processes, with deficiencies potentially leading to altered metabolic homeostasis and cell transformation (reviewed in refs (1,2)). Challenges to both nuclear and mitochondrial DNA stability arise not only spontaneously through processes of base deamination and loss, but also through exposure to various forms of radiation and other pro-oxidant environments, which are generated through electron-transport chains, certain biochemical pathways, and immune responses to bacterial and viral infections. Many of these genetic destabilizing events lead to free radical attacks on DNAs, with ionizing radiation being the most well characterized (reviewed in refs (1) and (2)). In the aqueous environment of nuclear and mitochondrial DNAs, exposure to free radicals generates a wide variety of products with the two most predominant lesions being 8-oxoguanine (8-oxo-Gua) and 2,6-diamino-4-hydroxy-5-formamidopyrimidine (FapyGua) (reviewed in refs (3) and (4)). As Figure 1 illustrates, 8-oxo-Gua and FapyGua are formed by one-electron oxidation and one-electron reduction of the C8-OH–adduct radical of guanine, respectively. This radical is generated by the addition of a hydroxyl radical to the C8-position of guanine in DNA (reviewed in refs (3,5−7)).

Both lesions are highly mutagenic and give rise to G→T transversion mutations,^8−12^ with FapyGua also leading to G→A transitions, G→C transversions, and single nucleotide deletions.^7,13^ Analyses of mutational events, including those at mutation hotspots in the *p53* gene, have revealed that FapyGua is more mutagenic than 8-oxo-Gua.^10,12−14^ 4,6-Diamino-5-formamidopyrimidine (FapyAde) is formed from adenine through a similar mechanism to that of FapyGua (reviewed in ref (3)). Given the biological consequences arising from the persistence of these lesions in DNA, cells possess dedicated repair mechanisms, primarily the base excision repair (BER) pathway to correct these damages. In this process, DNA glycosylases initiate the first step in the BER pathway by removing modified or damaged DNA bases, including those modified oxidatively (reviewed in refs (15−18)).

8-Oxoguanine DNA Glycosylase-1 (OGG1) is one of the major DNA glycosylases in mammals and other organisms. In a pioneering study, the *OGG1* gene of *Saccharomyces cerevisiae* was cloned and expressed, and the OGG1 protein (yOGG1) consisting of 376 amino acids isolated.^19^ The excision of 8-oxo-Gua from DNA was demonstrated, along with the release of 2,6- diamino-4-hydroxy-5-*N*-methylformamidopyrimidine, a methylated derivative of FapyGua from an oligodeoxynucleotide. Subsequently, the substrate specificity of yOGG1 was tested on four different genomic DNA substrates damaged by ionizing radiation- or H_2_O_2_/metal ion-generated free radicals,^20^ leading to the formation of DNA substrates with a plethora of pyrimidine- and purine-derived DNA base lesions. Gas chromatography-mass spectrometry with isotope dilution was used to measure these products and to study the enzyme-catalyzed excision kinetics of specific DNA base lesions. The results showed the ability of yOGG1 to excise 8-oxo-Gua and FapyGua from all four DNA substrates. Later, human α-OGG1 (α-hOGG1), its polymorphic variant α-hOGG1-Cys^326^, and its mutant forms α-hOGG1-Gln^46^ and α-hOGG1-His^154^ found in human tumors, along with *Drosophila melanogaster* OGG1, *Arabidopsis thaliana* OGG1, and natural polymorphic variants and phosphomimetic mutants of hOGG1 were isolated and purified.^21−26^ These proteins were tested using DNA substrates and methodologies similar to those in the previous study. All these OGG1 proteins efficiently excised 8-oxo-Gua and FapyGua with different excision kinetics. Unfortunately, despite the numerous published reports over many years and summarized above, with the exception of the investigation by Donley et al.,^27^ FapyGua has been largely neglected, with the primary focus remaining on 8-oxo-Gua.

**Figure 2:**
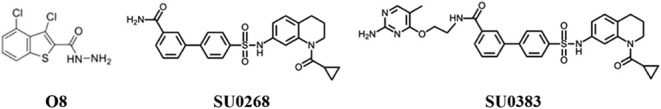
Structures of O8, SU0268, and SU0383 (Reprinted (adapted) with permission from Donley, N.; Jaruga, P.; Coskun, E.; Dizdaroglu, M.; McCullough, A. K.; Lloyd, R. S. Small Molecule Inhibitors of 8-Oxoguanine DNA Glycosylase-1 (OGG1). ACS Chem. Biol. 2015, 10 (10), 2334–2343. DOI: 10.1021/acschembio.5b00452). Copyright (2015) American Chemical Society, ref (27) and from Kant, M.; Tahara, Y. K.; Jaruga, P.; Coskun, E.; Lloyd, R. S.; Kool, E. T.; Dizdaroglu, M. Inhibition by tetrahydroquinoline sulfonamide derivatives of the activity of human 8-oxoguanine DNA glycosylase (OGG1) for several products of oxidatively induced DNA base lesions. *ACS Chem. Biol.***2021**, 16 (1), 45–51. DOI: 10.1021/acschembio.0c00877. Copyright (2021) American Chemical Society, ref (37).

Cancer cells increase their DNA repair capacity by overexpressing DNA repair proteins, leading to drug or radiation resistance in cancer therapy. Several inhibitors of DNA repair proteins have been approved or are under development as potential anticancer drugs (reviewed in refs. (28−30)) hOGG1 was one of the DNA glycosylases investigated as a therapeutic target for this purpose. Small molecule inhibitors of hOGG1 were developed and investigated by several laboratories.^27,31−38^ Following the screening of approximately 50000 compounds, Donley et al. identified five hydrazide derivatives with IC_50_ values in the range of 0.22 μmol/L to 0.63 μmol/L that efficiently inhibited the excision of 8-oxo-Gua and FapyGua by OGG1 from genomic DNA.^27^ These compounds were demonstrated to inhibit the combined glycosylase/lyase activity of OGG1. Figure 2 illustrates the structure of the compound O8 (3,4-dichlorobenzo[b]thiophene-2-carbohydrazide) with the highest IC_50_ among them. Tahara et al. screened approximately 26000 compounds and modified two selected ones with an acyl tetrahydroquinoline sulfonamide skeleton.^32^ These compounds 4′-(*N*-(1-(cyclopropanecarbonyl)-1,2,3,4-etrahydroquinolin-7-yl)-sulfamoyl)-[1,1′-biphenyl]-3-carboxamide (SU0268) and *N*-(2-((2-amino-5-methylpyrimidin-4-yl)oxy)ethyl)-4′-(*N*-(1-(cyclopropanecarbonyl)-1,2,3,4-tetrahydroquinolin-7-yl)sulfamoyl)-[1,1′-biphenyl]-3-carboxamide (SU0383) exhibited strong inhibition of 8-oxo-Gua excision by OGG1 using a fluorogenic assay.^32,39^Figure 2 illustrates the structures of these three compounds. Subsequently, SU0268 and SU0383 were also tested for inhibition of the excision of 8-oxo-Gua and FapyGua, and the minor substrate FapyAde by OGG1 from genomic DNA.^37^ Efficient inhibition of excision by these molecules was observed with SU0268 determined to be more potent than SU0383. None of these OGG1 inhibitors exhibited inhibition of other DNA glycosylases such as human NEIL1 and NTHL1, and *E. coli* Fpg.

OGG1 has also been shown to play an essential role in the initiating the innate immune inflammatory response via noncatalytic binding to oxidatively-induced DNA damage, presumably 8-oxo-Gua sites in promoter regions, followed by recruitment of activating transcription factors, eliciting gene-expression cascades.^40−45^ To explore drug-targeted strategies to blunt these inflammatory pathways, such as those observed in asthma pathogenesis, a synthetic site-specific oligodeoxynucleotide was developed forming a stable duplex, hairpin oligodeoxynucleotide containing a 8-OH-Ade opposite noncognate Cyt. Visnes et al.^33^ reported the discovery of a small molecule active-site inhibitor of hOGG1. Previously, yOGG1 and murine OGG1 had been shown to remove 8-OH-Ade from an oligodeoxynucleotide when paired with Cyt or 5-methyl-Cyt.^46,47^

The lead molecule identified by Visnes et al. was 4-(4-bromo-2-oxo-3H-benzimidazol-1-yl)-N-(4-iodophenyl)piperidine-1-carboxamide (TH5487) (structure is shown in Figure 4D) which had a reported half-maximal inhibitory concentration (IC_50_) of 0.342 μmol/L. TH5487 was found to prevent hOGG1 from binding to its substrate in DNA and to suppress proinflammatory gene expression and inflammation, demonstrating that the hOGG1 inhibition could be a potential strategy for the treatment of inflammation. Subsequent work by the Helleday group showed that inhibiting hOGG1 by TH5487 suppresses cancer cell growth, validating OGG1 as a potential anticancer target,^36^ and mitigates pulmonary inflammation and lung fibrosis.^38^ It also sensitizes cancer cells to radiation by high linear energy transfer protons.^48^ All these data confirmed hOGG1 as a promising target for pharmaceutical intervention. However, given that the oligodeoxynucleotide substrate with 8-OH-Ade opposite Cyt used for the drug-discovery portion of the study is not a primary substrate for OGG1, the present investigation was designed to determine the scope of hOGG1 substrates inhibited by TH5487. Our experimental approach differs significantly from those using site-specifically modified oligodeoxynucleotides with defined lesions and leveraging the biologically relevant substrate range of damaged genomic DNAs. This is achieved through gas chromatography-tandem mass spectrometry (GC-MS/MS) with isotope dilution, a methodology that accurately identifies and quantifies DNA base lesions removed or not removed from damaged genomic DNA by a given DNA glycosylase (reviewed in ref (17)). Using a genomic DNA substrate γ-irradiated at a low dose in aqueous solution, we provide the first direct measurement of the inhibition of the hOGG1-mediated efficient excision of 8-oxo-Gua and FapyGua by TH5487. Additionally, we detected inhibition of the release of FapyAde confirming this activity of hOGG1 as previously reported.^37^

**Figure 3:**
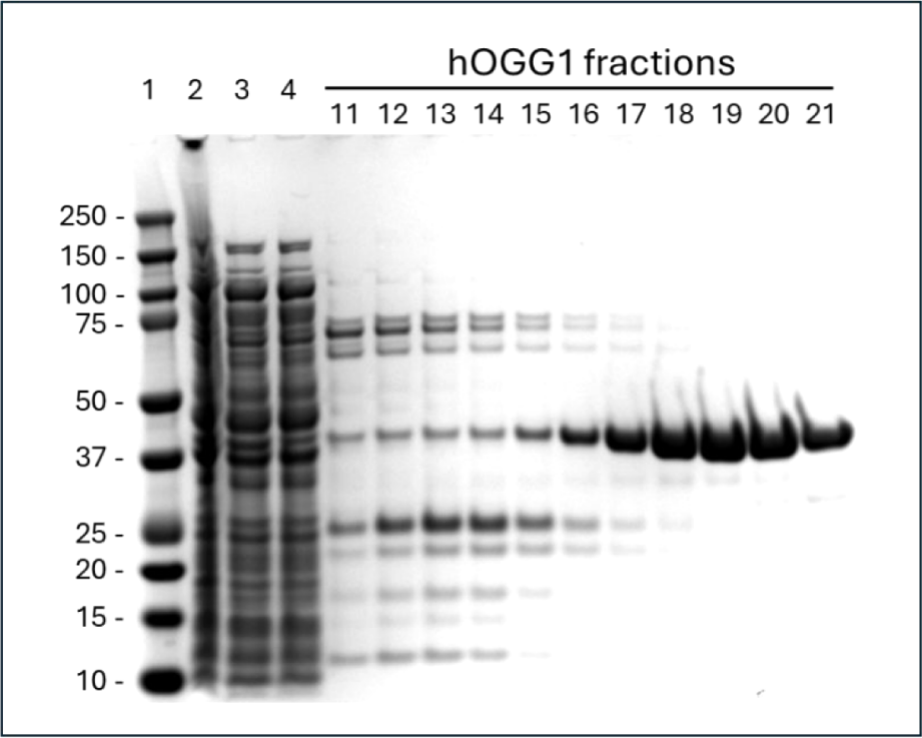
Coomassie blue visualization of the purification of hOGG1. Lane 1, molecular weight markers (Precision Plus Protin Dual Color Standards (Bio-Rad)); lane 2, *E. coli* pellet before pre-French press breakage; lane 3, load; lane 4, flow-through of the loaded proteins; and lanes 5–15, aliquots of fractions 11–21. Fractions 19–21 were combined, for dialysis into storage buffer, aliquoted, and stored at −80 °C.

## Materials and Methods

### Materials

TH5487 was purchased from Sigma-Aldrich Chemical Co. (St. Louis, MO, USA). The nickel-resin matrix (HisPur Ni-NTA Resin) for purification of OGG1 was purchased from Thermo Fisher Scientific (Waltham, MA, USA). Synthetic site-specifically modified oligodeoxynucleotides were generously provided by Dr. Carmelo Rizzo, Department of Chemistry, Vanderbilt University, Nashville, TN, USA. The complementary black hole quencher 2 (BHQ2)-labeled oligodeoxynucleotide was purchased from Integrated DNA Technologies (Coralville, IA, San Diego, CA, USA). Kinetic assays were performed using black, flat bottom, low flange 384 well plates purchased from Corning Inc. (Corning, NY, USA). All other common chemical reagents were purchased from Thermo Fisher Scientific (Waltham, MA, USA). Human NTHL1 protein was a gift from Prof. Joann Sweasy of the University of Arizona, Tucson, AZ, USA. Cloning, expression, and purification of human NEIL1 protein were performed as described previously.^49^

### Purification of hOgg1

The vector for the expression of hOGG1 was engineered to encode a N-terminal 6-His affinity purification tag and introduced into BL21(DE3) *E. coli* for expression (New England Biolabs, Ipswich, MA, USA). Individual clones were obtained on lysogeny broth (LB)-agar plates containing 50 μg/mL ampicillin. Colonies were inoculated into 20 mL LB media (1 % tryptone (v/v), 0.5 % yeast extract (v/v), 1 % NaCl (w/v), pH 7.0) containing 50 μg/mL ampicillin and grown overnight at 37 °C. The overnight culture was used to inoculate 2 L of LB media and the culture was incubated at 37 °C until the OD_600_ reached 0.8. Expression of OGG1 and mutants was induced by the addition of isopropyl 1-thio-β-D-galactopyranoside to a final concentration of 0.5 mmol/L and incubation continued for 6 h at 20 °C. Cells were pelleted by centrifugation at ≈ 4000× *g* for 10 min and stored at −80 °C. The frozen pellet was resuspended to a single-cell suspension on ice in a binding buffer for Ni^2+^ chromatography (50 mmol/L sodium phosphate, pH 8), 300 mmol/L sodium chloride, 50 mol/L imidazole, 5 mmol/L β-mercaptoethanol), followed by lysis using a French pressure cell at 96.5 MPa (14000 psi), and brief sonication (3 times 10 s each). Cellular debris was removed by centrifugation at ≈ 22000 × *g* for 20 min, and the supernatant was loaded onto a 10 mL Qiagen Ni-NTA agarose column that was pre-equilibrated with binding buffer. The column was washed with ≈ 25 column volumes using binding buffer until the optical density at 280 nm was <0.05. hOGG1 was eluted with 150 mL binding buffer containing a gradient of 50 mol/L to 500 mmol/L imidazole. Fractions (3 mL) were collected, and the protein concentration of each fraction was measured with the Bradford reagent (Bio-Rad) using an Infinite M200 Tecan system. The purity of hOGG1 in each fraction was evaluated by gel electrophoresis with a NuPAGE 4 % to 12 % Bis-Tris gel under denaturing conditions. Gels were stained with Bio-Safe Coomassie Blue (Bio-Rad Laboratories, Hercules, CA, USA) (Figure 3). Fractions containing the purest hOGG1 (fractions 19-21) were pooled and dialyzed against 50 mmol/L Tris-HCl, pH 7.4, 150 mmol/L sodium chloride, 0.1 mmol/L ethylenediaminetetraacetic acid (EDTA), and 10 % (v/v) glycerol. Aliquots were flash-frozen and stored at −80 °C. To estimate the percentage of active OGG1 molecules in the final preparation, oligodeoxynucleotide nicking assays were performed to measure the combined glycosylase/AP lyase over a two-order magnitude range of enzyme concentrations assessing both glycosylase and AP lyase activities. Analyses of the data revealed that approximately 10% of the reactions carried out both the glycosylase and AP lyase steps. When the same experiments were conducted in the presence of excess human apurinic/apyrimidinic endonuclease1 (APE1), the percent product formed significantly increased by approximately 3-fold. Assuming that the addition of APE1 did not affect the rate of the glycosylase step or alter enzyme turnover, it was estimated that the final preparation of OGG1 is 30 % active.

### Duplex Oligodeoxynucleotide Incision Assay

Inhibition of hOGG1 by TH5487 was determined using a fluorescence-based assay oligodeoxynucleotide cleavage with concentrations of TH5487 ranging from 0.024 μmol/L to 25 μmol/L. A 17-mer 8-oxo-Gua–containing oligodeoxynucleotide with a 5-carboxytetramethylrhodamine (5-TAMRA) fluorophore and a complementary oligodeoxynucleotide with a 3′-BHQ2 were combined at a 1:1.2 ratio in 20 mmol/L Tris (pH 7.4), 100 mmol/L KCl, and 0.01 % Tween-20 and annealed by heating to 95 °C for 2 min followed by slow cooling to 4 °C. Reactions were performed at 37 °C in a reaction volume of 20 μL. hOGG1 was diluted to 500 nmol/L in a buffer containing 20 mmol/L Tris (pH 7.4), 100 mmol/L KCl, 0.01% Tween-20, and 100 μg/mL bovine serum albumin (BSA). TH5487 was dissolved in 100% dimethyl sulfoxide (DMSO). For each reaction, 32 μL of 500 nmol/L hOGG1 was premixed with 8 μL of various concentrations of TH5487. Reactions were initiated by adding 10 μL of the OGG1 + TH5487 mixture to 10 μL of 100 nmol/L 8-oxo-Gua–containing oligodeoxynucleotides in a 384-well black-bottom microplate using a multichannel pipet. The final concentrations of reactants in all reactions were as follows: 200 nmol/L hOGG1, 50 nmol/L 8-oxo-Gua–containing oligodeoxynucleotide, 0.024 μmol/L to 25 μmol/L TH5487, and 10 % (v/v) DMSO. Fluorescence readings were taken every two min for 1 h at 37 °C in a TECAN INFINITE M NANO instrument using a 525 nm (9 nm bandwidth) excitation filter and a 598 nm (20 nm bandwidth) emission filter. All reactions were performed in technical duplicate. Readings from technical duplicates were averaged, and the initial rate was determined by fitting data from 2 min to 40 min of the reaction to linear function using Excel. The initial rate was divided by that of the OGG1 + DMSO control reaction to calculate relative activity. The IC_50_ concentration was calculated by fitting a nonlinear regression to the linear reaction rates derived from each TH5487 concentration. These calculations were performed using ATT Bioquest (https://www.aatbio.com/tools/ic50-calculator).

### Analysis of DNA Lesions by GC-MS/MS with Isotope-Dilution

Calf thymus DNA was γ-irradiated in a ^60^Co-γ source in N_2_O-saturated buffered aqueous solution at a dose of 10 Gy and then dialyzed as described previously.^50^ Aliquots of 50 μg of DNA samples were dried in a SpeedVac. For each data point, a triplicate of 50 μg of DNA samples were supplemented with aliquots of 8-oxo-Gua-^15^N_5_, FapyGua-^13^C,^15^N_2_ and FapyAde-^13^C,^15^N_2_ as internal standards.^37^ The samples were then dissolved in 50 μL of an incubation buffer consisting of 50 mmol/L phosphate buffer (pH 7.4), 100 mmol/L KCl, 1 mmol/L EDTA, and 0.1 mmol/L dithiothreitol. TH5487 was dissolved in DMSO at a concentration of 10 mmol/L, which provided a complete solubility. The aliquots of these solutions were added to the reaction mixture to achieve the desired final concentration of the inhibitors. This was followed by incubation with 49 pmol of hOGG1 (0.97 μmol/L) at 37 °C for 1 h to release the modified DNA bases from DNA. Control samples added DMSO only. An aliquot of 100 μL ethanol was added to precipitate DNA and to stop the reaction. After centrifugation, the supernatant fractions were separated, lyophilized, derivatized by trimethylsilylation, and then analyzed by GC-MS/MS using multiple reaction monitoring (MRM) as described previously.^51−53^ The following mass/charge (*m*/*z*) transitions were used for the identification and quantification: *m*/*z* 455 → *m*/*z* 440 and *m*/*z* 460 → *m*/*z* 445 for 8-oxo-Gua and 8-oxo-Gua-^15^N_5_, respectively; *m*/*z* 457 → *m*/*z* 368 and *m*/*z* 460 → *m*/*z* 371 for FapyGua and FapyGua-^13^C,^15^N_2_, respectively; *m*/*z* 369 → *m*/*z* 368 and *m*/*z* 372 → *m*/*z* 371 for FapyAde and FapyAde-^13^C,^15^N_2_, respectively.

### Statistical Analysis

Three independently prepared DNA samples were used for each data point shown. Statistical analyses of the data were performed by using the GraphPad Prism 6 software (La Jolla, CA, USA) and unpaired, two-tailed nonparametric Mann–Whitney test with Gaussian approximation and a confidence of 99%.

## Results and Discussion

### Inhibition of hOgg1 Incision on Duplex Oligodeoxynucleotide Containing an 8-Oxo-Gua Opposite Cyt

**Figure 4:**
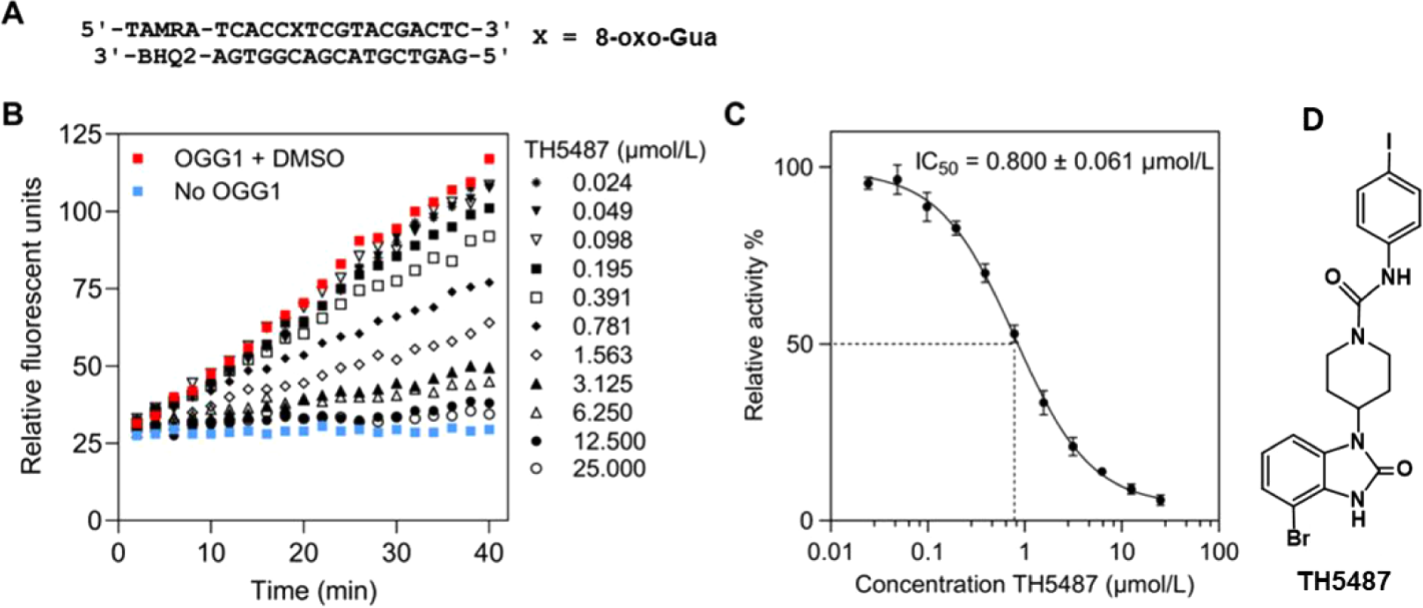
TH5487 inhibition of hOGG1 activity on 8-oxo-Gua–containing oligodeoxynucleotide substrate. (A) Sequence of 8-oxo-Gua–containing oligodeoxynucleotide substrate. (B) Data showing TH5487 inhibition of hOGG1 activity. Increasing concentrations of TH5487 or DMSO was mixed with 16 pmol of hOGG1 and added to the 8-oxo-Gua–containg oligodeoxynucleotide substrate. Fluorescence was measured every 2 min using a Tecan plate reader. (C) IC_50_ determination for TH5487 inhibition of hOGG1 activity on the 8-oxo-Gua–containing oligodeoxynucleotide substrate. The IC_50_ of TH5487 was calculated as described in Materials and Methods. Data presented in Figure 4C reflects three independent experiments. Uncertainties indicate standard deviations. (D) Structure of TH5487.

Although the inhibition of hOGG1 by TH5487 has been inferred from the loss of catalytic efficiency in studies using an 8-OH-Ade:Cyt hairpin substrate,^33^ this inhibition has not been directly assayed on the primary biological substrates of hOGG1, which are 8-oxo-Gua and FapyGua (*vide supra*). In the case of the 8-OH-Ade:Cyt substrate, the previously reported IC_50_ was 0.342 μmol/L, measured in the presence of APE1. Using commercially available TH5487, we independently established an IC_50_ value using a duplex oligodeoxynucleotide containing a centrally located, site-specific 8-oxo-Gua:Cyt pair (Figure 4A). The fluorescence-based design of this duplex, previously reported,^27^ relies on both the glycosylase and AP lyase activities of hOGG1, incising the TAMRA-labeled strand and releasing it into free solution. Inhibition of hOGG1 activity by TH5487 in this assay was shown to be concentration-dependent (Figure 4B), with data from three independent experiments establishing an IC_50_ of 0.800 μmol/L ± 0.061 μmol/L (Figure 4C). The structure of TH5487 is shown in Figure 4D.

### Inhibition of hOgg1-Mediated Release of Oxidatively Induced Genomic DNA Damage by TH5487

**Figure 5:**
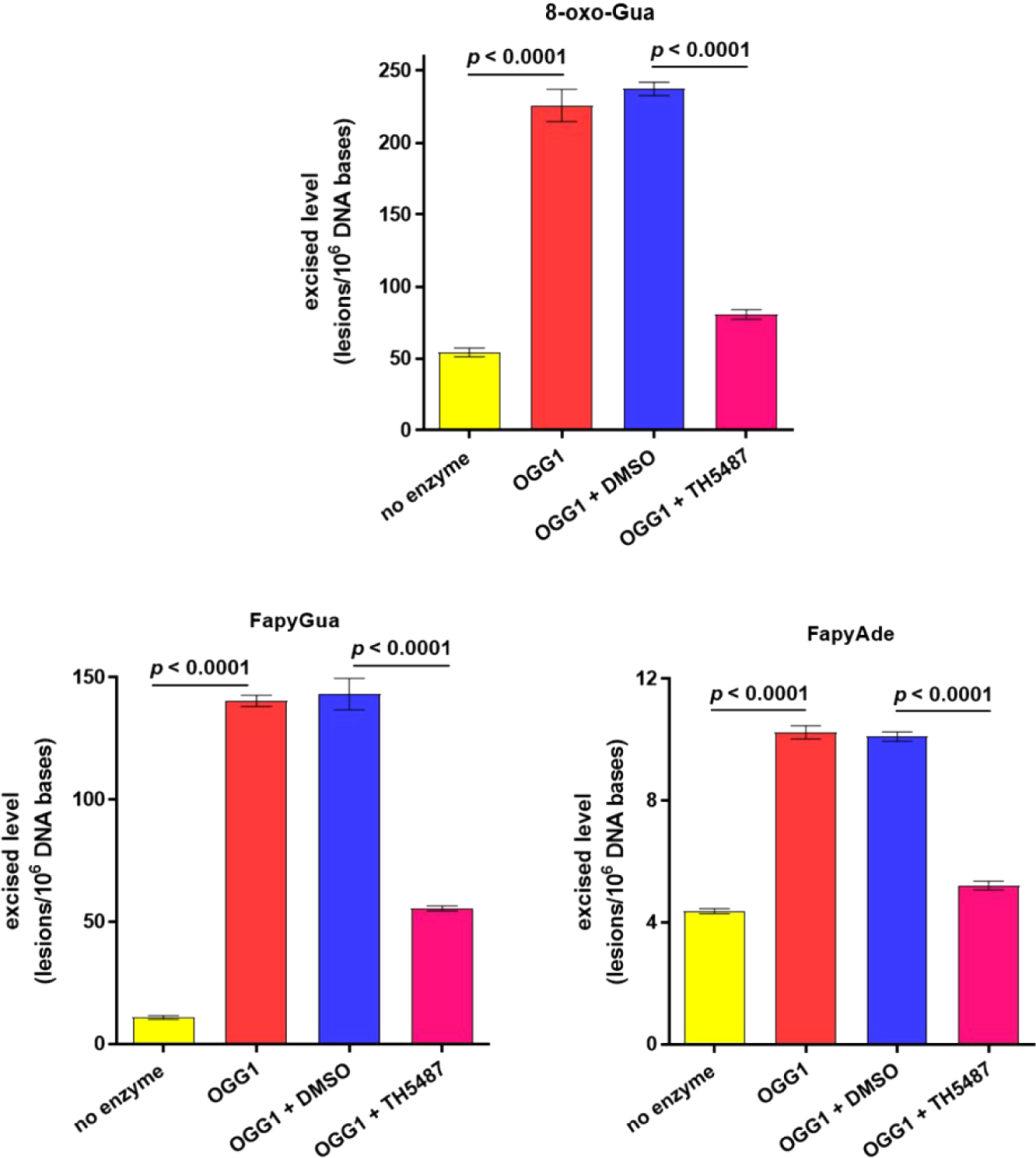
Excised levels of 8-oxo-Gua, FapyGua, and FapyAde from DNA by hOGG1, hOGG1 plus DMSO, and hOGG1 with TH5487 are shown at a concentration of 10 μmol/L. Control levels without hOGG1 are also included. Uncertainties are standard deviations. The *p*-values < 0.05 indicate statistical significance.

A concentration of 10 μmol/L of TH5487 was initially used to test for the inhibition of hOGG1 on genomic DNA containing a variety of purine- and pyrimidine-derived lesions. This concentration of TH5487 was reported to be well tolerated in cultured human cells.^33^ In addition to 8-oxo-Gua and FapyGua, we also tested the excision of FapyAde, which had been shown to be a minor substrate of hOGG1,^37^ although it is one of the main substrates of *E. coli* Fpg, the bacterial analogue of hOGG1.^54,55^ The control levels of 8-oxo-Gua, FapyGua, and FapyAde as well as the levels excised by hOGG1, hOGG1 plus DMSO and hOGG1 plus TH5487 at a concentration of 10 μmol/L are shown in Figure 5. hOGG1 with DMSO alone was also tested as the inhibitor was dissolved in DMSO. No significant effect of DMSO on the excisions was observed. The *p*-values in Figure 5 show the significance of the inhibition of hOGG1 by TH5487 for the three lesions. Inhibition by TH5487 was over 60 % for 8-oxo-Gua and FapyGua. In the case of FapyAde, the excision was almost inhibited to the control level.

**Figure 6:**
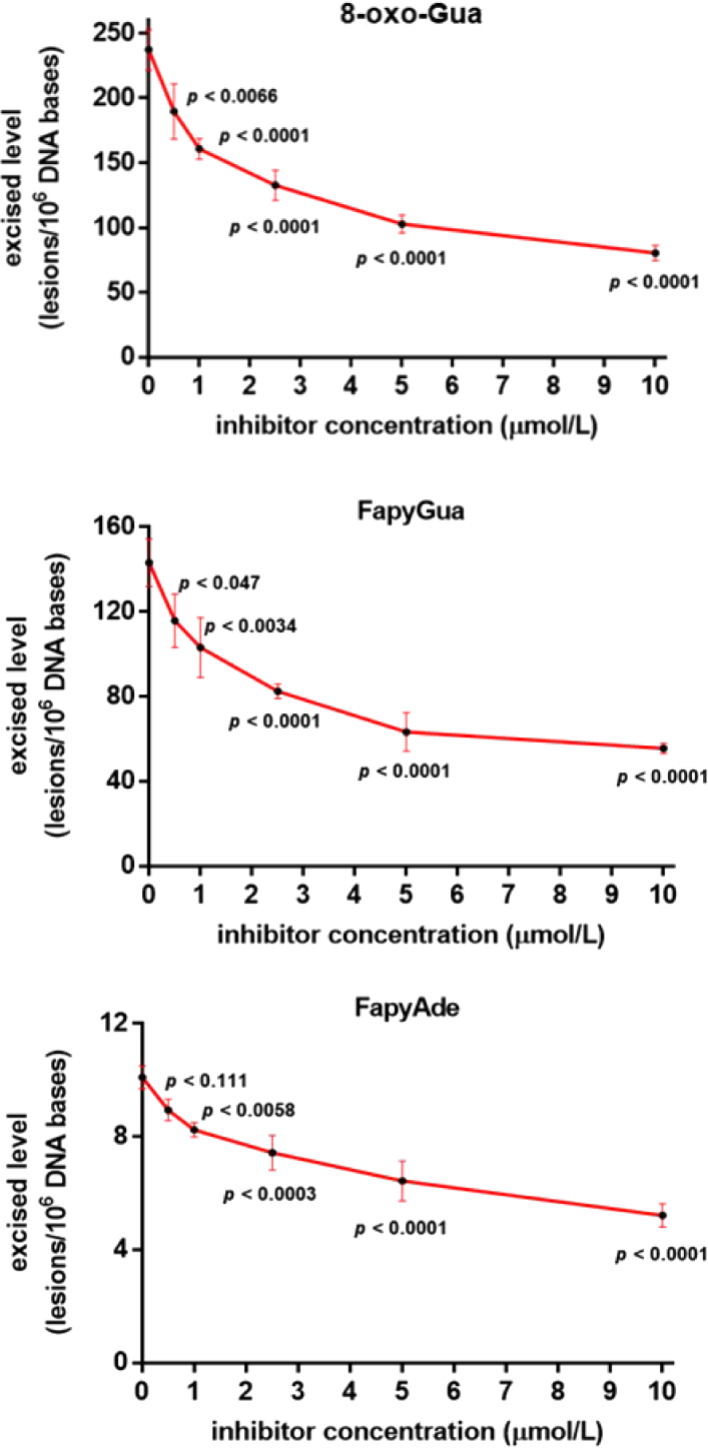
Dependence of the excised levels of 8-oxo-Gua, FapyGua, and FapyAde on the concentration of TH5487. Uncertainties are standard deviations. The *p*-values < 0.05 show the statistical significance between the excised levels with TH5487 and without TH5487.

Next, inhibition was tested at increasing concentrations of TH5487 ranging from 0.5 μmol/L to 10 μmol/L. The concentration-dependent inhibition by TH5487 is shown in Figure 6.

The excised levels of the three substrates decreased with increasing amounts of the inhibitor. The *p*-values indicate the significance between the excision without TH5487 and with increasing concentrations of TH5487. We also tested human DNA glycosylases NEIL1 and NTHL1 to determine whether TH5487 also inhibits the excision of substrates of these proteins, including FapyGua and FapyAde, but not 8-oxo-Gua, which is not a substrate of these proteins (reviewed in ref (17)). No inhibition by TH5487 of these substrates or any other substrates of human NEIL1 and NTHL1 was observed (data not shown).

**Figure 7:**
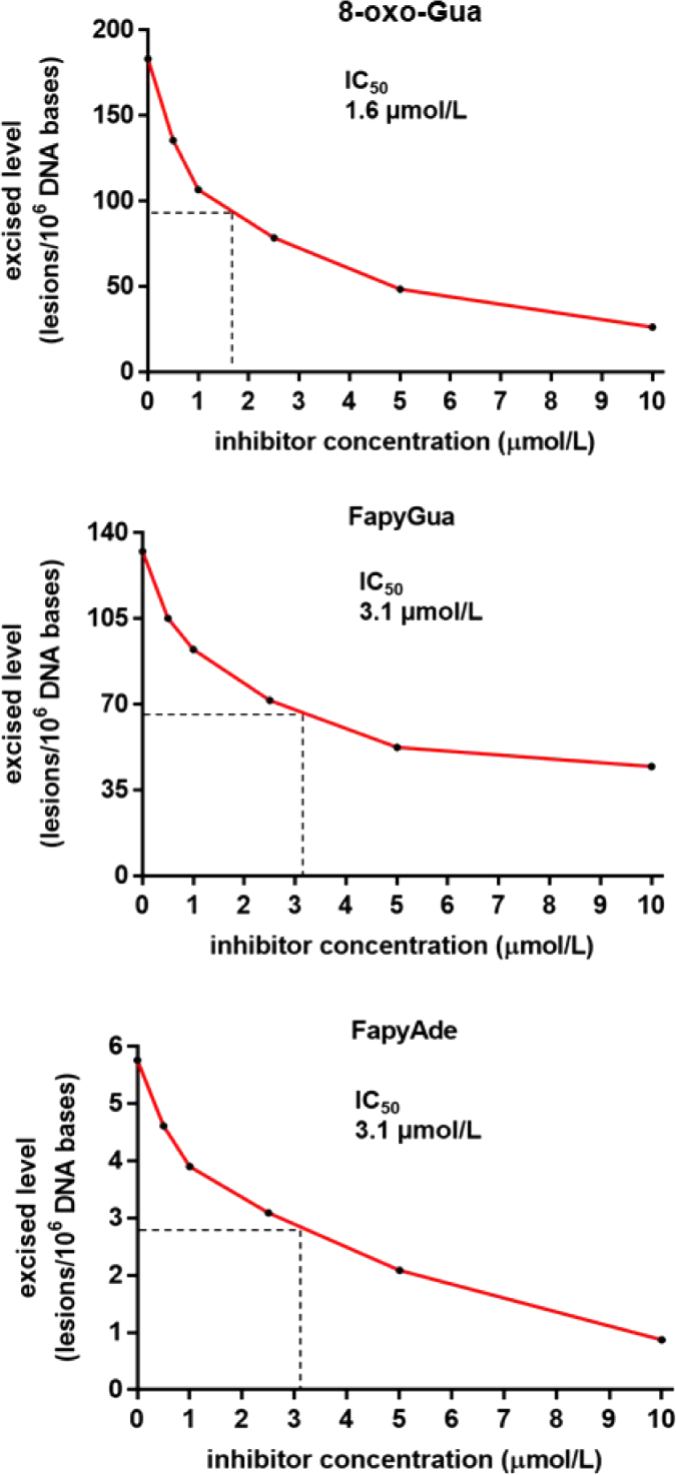
Dependence of the background-subtracted excised levels of 8-oxo-Gua, FapyGua, and FapyAde on the concentration of TH5487 with the estimated IC_50_ values.

Based on the data presented in Figure 6, we attempted to determine the IC_50_ value for each substrate as IC_50_ values are generally determined using short oligodeoxynucleotides containing a single DNA base lesion such as in Figure 4. For this purpose, we first subtracted the background (no enzyme) values of each lesion shown in Figure 5 from the values in Figure 6, as the background-subtracted values represent the levels of the substrates removed by the enzyme only. These data are replotted in Figure 7. The IC_50_ values were determined at 50 % of the excised levels. The IC_50_ value of 8-oxo-Gua at 1.6 μmol/L is twice greater than that observed with the 17-mer 8-oxo-Gua-containing oligodeoxynucleotide (Figure 4). The observed difference may be attributed to the varying size of substrates, with one being a 17-mer oligodeoxynucleotide containing a single lesion and the other one being high molecular-weight DNA containing multiple pyrimidine- and purine-derived lesions. Interestingly, the IC_50_ values for the chemically similar compounds FapyGua and FapyAde were equal at 3.1 μmol/L. The IC_50_ values for these substrates in the context of TH5487 inhibition have not been measured previously.

## Conclusions

Our data show for the first time the potent inhibition of hOGG1 by TH5487 for the excision of 8-oxo-Gua from genomic DNA using a methodology which is different from those previously used relative to the DNA substrates, the experimental design, the methodology and the measurement technique. Importantly, we also demonstrate that TH5487 equally inhibits the excision of FapyGua, another primary substrate of hOGG1 and other eukaryotic OGG1s (*vide supra*), as well as the excision of the minor substrate FapyAde. The inhibition of the hOGG1 excision of FapyGua or FapyAde by TH5487 has never been documented previously. Moreover, prior studies using TH5487 have not addressed this important substrate of hOGG1, which possesses mutagenic effects comparable to or even more frequent than those of 8-oxo-Gua (*vide supra*). The present work using a unique methodology provides a comprehensive analysis of the inhibition of hOGG1 by TH5487 for its physiological substrates. Considering that TH5487 may become a potentially important compound for the treatment of pulmonary inflammation, lung fibrosis and cancer,^33,36,38,48^ it would be important to include the inhibition of not only 8-oxo-Gua, but also that of FapyGua and FapyAde by hOGG1 in future studies to provide a broad basis for understanding the biological effects of this molecule. The same approach should be applied for other known or potential inhibitors of DNA glycosylases as they might be candidates for future clinical trials. Our methodology allows the simultaneous measurement of DNA base lesions released from genome by DNA glycosylases. It has enabled the discovery of the inhibition of additional biological substrates of hOGG1 by TH5487 and previously by other potent OGG1 inhibitors such as hydrazide derivatives and tetrahydroquinoline compounds, which may become clinically relevant drug candidates.^27,32,35,37^ The results of this and previous studies suggest that the methodology used in this work should be applied to future studies of TH5487 and other OGG1 inhibitors in cellular and animal model systems.

## Dedication

P.J., M.K., M.M.L., R.S.L., and I.B. dedicate this work to the memory of the exceptional scientist, NIST Fellow Dr. Miral Dizdaroglu, who sadly passed away on Dec 13th, 2024, during the last days of this article’s accomplishment. His insightful guidance, which was vital to this project, and his impactful work in the field of DNA damage and repair continue to inspire us and will inspire future generations. We are deeply grateful for the friendship and contributions of Dr. Dizdaroglu, one of the world’s leading scientists.

## References

[ref1] von Sonntag C. Free-Radical-Induced DNA Damage and Its Repair; Springer, 2006.

[ref2] Halliwell B.; Gutteridge J. M. C. Free Radicals in Biology & Medicine; Oxford University Press, 2015.

[ref3] Dizdaroglu M.; Jaruga P. Mechanisms of Free Radical-Induced Damage to DNA. Free Radical Res. 2012, 46 (4), 382–419. 10.3109/10715762.2011.653969. 22276778

[ref4] Halliwell B.; Adhikary A.; Dingfelder M.; Dizdaroglu M. Hydroxyl Radical is a Significant Player in Oxidative DNA Damage in Vivo. Chem. Soc. Rev. 2021, 50 (15), 8355–8360. 10.1039/D1CS00044F. 34128512 PMC8328964

[ref5] Steenken S. Purine bases, nucleosides, and nucleotides: aqueous solution redox chemistry and transformation reactions of their radical cations and e- and OH adducts. Chem. Rev 1989, 89 (3), 503–520. 10.1021/cr00093a003.

[ref6] Dizdaroglu M.; Kirkali G.; Jaruga P. Formamidopyrimidines in DNA: Mechanisms of Formation, Repair, and Biological Effects. Free Radical Biol. Med. 2008, 45 (12), 1610–1621. 10.1016/j.freeradbiomed.2008.07.004. 18692130

[ref7] Greenberg M. M. The Formamidopyrimidines: Purine Lesions Formed in Competition with 8-Oxopurines from Oxidative Stress. Acc. Chem. Res. 2012, 45 (4), 588–597. 10.1021/ar2002182. 22077696 PMC3292677

[ref8] Kuchino Y.; Mori F.; Kasai H.; Inoue H.; Iwai S.; Miura K.; Ohtsuka E.; Nishimura S. Misreading of DNA Templates Containing 8-Hydroxydeoxyguanosine at the Modified Base and at Adjacent Residues. Nature 1987, 327 (6117), 77–79. 10.1038/327077a0. 3574469

[ref9] Wood M. L.; Dizdaroglu M.; Gajewski E.; Essigmann J. M. Mechanistic Studies of Ionizing-Radiation and Oxidative Mutagenesis - Genetic-Effects of a Single 8-Hydroxyguanine (7-hydro-8-oxoguanine) Residue Inserted at a Unique Site in a Viral Genome. Biochemistry 1990, 29 (30), 7024–7032. 10.1021/bi00482a011. 2223758

[ref10] Kalam M. A.; Haraguchi K.; Chandani S.; Loechler E. L.; Moriya M.; Greenberg M. M.; Basu A. K. Genetic Effects of Oxidative DNA Damages: Comparative Mutagenesis of the Imidazole Ring-Opened Formamidopyrimidines (Fapy Lesions) and 8-Oxo-purines in Simian Kidney Cells. Nucleic Acids Res. 2006, 34 (8), 2305–2315. 10.1093/nar/gkl099. 16679449 PMC1458282

[ref11] Gao S.; Oden P. N.; Ryan B. J.; Yang H.; Freudenthal B. D.; Greenberg M. M. Biochemical and Structural Characterization of Fapy•dG Replication by Human DNA Polymerase β. Nucleic Acids Res. 2024, 52, 5392–5405. 10.1093/nar/gkae277. 38634780 PMC11109955

[ref12] Gao S.; Tahara Y.; Kool E. T.; Greenberg M. M. Promoter Dependent RNA Polymerase II Bypass of the Epimerizable DNA Lesion, Fapy.dG and 8-Oxo-2’-deoxyguanosine. Nucleic Acids Res. 2024, 52, 7437–7446. 10.1093/nar/gkae529. 38908029 PMC11260475

[ref13] Bacurio J. H. T.; Yang H.; Naldiga S.; Powell B. V.; Ryan B. J.; Freudenthal B. D.; Greenberg M. M.; Basu A. K. Sequence Context Effects of Replication of Fapy.dG in Three Mutational Hot Spot Sequences of the p53 Gene in Human Cells. DNA Repair 2021, 108, 103213 10.1016/j.dnarep.2021.103213. 34464900 PMC8616820

[ref14] Stanio S.; Bacurio J. H. T.; Yang H.; Greenberg M. M.; Basu A. K. 8-Oxo-2’-deoxyguanosine Replication in Mutational Hot Spot Sequences of the p53 Gene in Human Cells Is Less Mutagenic than that of the Corresponding Formamidopyrimidine. Chem. Res. Toxicol. 2023, 36 (5), 782–789. 10.1021/acs.chemrestox.3c00069. 37093780 PMC10192040

[ref15] Friedberg E. C.; Walker G. C.; Siede W.; Wood R. D.; Schultz R. A.; Ellenberger T. DNA Repair and Mutagenesis; ASM Press, 2006.

[ref16] Wallace S. S. Base excision repair: A Critical Player in Many Games. DNA Repair 2014, 19, 14–26. 10.1016/j.dnarep.2014.03.030. 24780558 PMC4100245

[ref17] Dizdaroglu M.; Coskun E.; Jaruga P. Repair of Oxidatively Iinduced DNA Damage by DNA Glycosylases: Mechanisms of Action, Substrate Specificities and Excision Kinetics. Mutat. Res., Rev. Mutat. Res. 2017, 771, 99–127. 10.1016/j.mrrev.2017.02.001. 28342455 PMC7451025

[ref18] Kant M.; Quintana V.; Coskun E.; Jaruga P.; Lloyd R. S.; Sweasy J. B.; Dizdaroglu M. Polymorphic Variant Asp239Tyr of Human DNA Glycosylase NTHL1 is Inactive for Removal of a Variety of Oxidatively-Induced DNA Base lLesions from Genomic DNA. DNA Repair 2022, 117, 103372 10.1016/j.dnarep.2022.103372. 35870279 PMC12459669

[ref19] van der Kemp P. A.; Thomas D.; Barbey R.; de Oliveira R.; Boiteux S. Cloning and Expression in Escherichia coli of the *OGG1* Gene of *Saccharomyces cerevisiae*, which Codes for a DNA glycosylase that excises 7,8-dihydro-8-oxoguanine and 2,6-diamino-4-hydroxy-5-N-Methylformamidopyrimidine. Proc. Natl. Acad. Sci. U. S. A. 1996, 93 (11), 5197–5202. 10.1073/pnas.93.11.5197. 8643552 PMC39221

[ref20] Karahalil B.; Girard P. M.; Boiteux S.; Dizdaroglu M. Substrate Specificity of the Ogg1 Protein of *Saccharomyces cerevisiae*: Excision of Guanine Lesions Produced in DNA by Ionizing Radiation- or Hydrogen Peroxide/Metal Ion-generated Free Radicals. Nucleic Acids Res. 1998, 26 (5), 1228–1233. 10.1093/nar/26.5.1228. 9469830 PMC147376

[ref21] Dherin C.; Radicella J. P.; Dizdaroglu M.; Boiteux S. Excision of Oxidatively Damaged DNA Bases by the Human Alpha-hOgg1 Protein and the Polymorphic Alpha-hOgg1(Ser326Cys) Protein which is Frequently Found in Human Populations. Nucleic Acids Res. 1999, 27 (20), 4001–4007. 10.1093/nar/27.20.4001. 10497264 PMC148667

[ref22] Audebert M.; Radicella J. P.; Dizdaroglu M. Effect of Single Mutations in the OGG1 Gene Found in Human Tumors on the Substrate Specificity of the Ogg1 Protein. Nucleic Acids Res. 2000, 28 (14), 2672–2678. 10.1093/nar/28.14.2672. 10908322 PMC102664

[ref23] Dherin C.; Dizdaroglu M.; Doerflinger H.; Boiteux S.; Radicella J. P. Repair of Oxidative DNA Damage in *Drosophila melanogaster*: Identification and Characterization of dOgg1, a Second DNA Glycosylase Activity for 8-Hydroxyguanine and Formamidopyrimidines. Nucleic Acids Res. 2000, 28 (23), 4583–4592. 10.1093/nar/28.23.4583. 11095666 PMC115177

[ref24] Garcia-Ortiz M. V.; Ariza R. R.; Roldan-Arjona T. An OGG1 Orthologue Encoding a Functional 8-Oxoguanine DNA Glycosylase/Lyase in Arabidopsis Thaliana. Plant Mol. Biol. 2001, 47 (6), 795–804. 10.1023/A:1013644026132. 11785940

[ref25] Morales-Ruiz T.; Birincioglu M.; Jaruga P.; Rodriguez H.; Roldan-Arjona T.; Dizdaroglu M. *Arabidopsis Thaliana* Ogg1 Protein Excises 8-Hydroxyguanine and 2,6-Diamino-4-Hydroxy-5-Formamidopyrimidine from Oxidatively Damaged DNA Containing Multiple Lesions. Biochemistry 2003, 42 (10), 3089–3095. 10.1021/bi027226u. 12627976

[ref26] Sidorenko V. S.; Grollman A. P.; Jaruga P.; Dizdaroglu M.; Zharkov D. O. Substrate Specificity and Excision Kinetics of Natural Polymorphic Variants and Phosphomimetic Mutants of Human 8-Oxoguanine-DNA Glycosylase. Febs J. 2009, 276 (18), 5149–5162. 10.1111/j.1742-4658.2009.07212.x. 19674107 PMC2746928

[ref27] Donley N.; Jaruga P.; Coskun E.; Dizdaroglu M.; McCullough A. K.; Lloyd R. S. Small Molecule Inhibitors of 8-Oxoguanine DNA Glycosylase-1 (OGG1). ACS Chem. Biol. 2015, 10 (10), 2334–2343. 10.1021/acschembio.5b00452. 26218629 PMC4894821

[ref28] Curtin N. J. The Role of PARP and the Therapeutic Potential of PARP Inhibitors in Cancer. In DNA Damage, DNA Repair and Disease, Dizdaroglu M.; Lloyd R. S., Eds.; Royal Society of Chemistry, 2021, pp. 319–360.

[ref29] Sadiq M. T.; Madhusudan S. Evolving DNA Repair Targets for Cancer Therapy. In DNA Damage, DNA Repair and Disease, Vol., Dizdaroglu M.; Lloyd R. S., Eds.; Royal Society of Chemistry, 2021, pp. 254–285.

[ref30] Gampala S.; Caston R. A.; Fishel M. L.; Kelley M. R. Basic Translational and Clinical Relevance of the DNA Repair and Redox Signaling Protein APE1. In Human Diseases In DNA Damage, DNA Repair and Disease Vol., Dizdaroglu M.; Lloyd R. S., Eds.; Royal Society of Chemistry, 2021, pp. 286–318.

[ref31] Jacobs A. C.; Calkins M. J.; Jadhav A.; Dorjsuren D.; Maloney D.; Simeonov A.; Jaruga P.; Dizdaroglu M.; McCullough A. K.; Lloyd R. S. Inhibition of DNA Glycosylases Via Small Molecule Purine Analogs. PLoS One 2013, 8 (12), e81667 10.1371/journal.pone.0081667. 24349107 PMC3857224

[ref32] Tahara Y. K.; Auld D.; Ji D.; Beharry A. A.; Kietrys A. M.; Wilson D. L.; Jimenez M.; King D.; Nguyen Z.; Kool E. T. Potent and Selective Inhibitors of 8-Oxoguanine DNA Glycosylase. J. Am. Chem. Soc. 2018, 140 (6), 2105–2114. 10.1021/jacs.7b09316. 29376367 PMC5823510

[ref33] Visnes T.; Cázares-Körner A.; Hao W.; Wallner O.; Masuyer G.; Loseva O.; Mortusewicz O.; Wiita E.; Sarno A.; Manoilov A.; et al. Small-Molecule Inhibitor of OGG1 Suppresses Proinflammatory Gene Expression and Inflammation. Science 2018, 362 (6416), 834–839. 10.1126/science.aar8048. 30442810 PMC6645780

[ref34] Visnes T.; Grube M.; Hanna B. M. F.; Benitez-Buelga C.; Cazares-Korner A.; Helleday T. Targeting BER enzymes in cancer therapy. DNA Repair 2018, 71, 118–126. 10.1016/j.dnarep.2018.08.015. 30228084

[ref35] Tahara Y. K.; Kietrys A. M.; Hebenbrock M.; Lee Y.; Wilson D. L.; Kool E. T. Dual Inhibitors of 8-Oxoguanine Surveillance by OGG1 and NUDT1. ACS Chem. Biol. 2019, 14 (12), 2606–2615. 10.1021/acschembio.9b00490. 31622553 PMC7061906

[ref36] Visnes T.; Benitez-Buelga C.; Cázares-Körner A.; Sanjiv K.; Hanna B. M. F.; Mortusewicz O.; Rajagopal V.; Albers J. J.; Hagey D. W.; Bekkhus T.; et al. Targeting OGG1 arrests cancer cell proliferation by inducing replication stress. Nucleic Acids Res. 2020, 48 (21), 12234–12251. 10.1093/nar/gkaa1048. 33211885 PMC7708037

[ref37] Kant M.; Tahara Y. K.; Jaruga P.; Coskun E.; Lloyd R. S.; Kool E. T.; Dizdaroglu M. Inhibition by tetrahydroquinoline sulfonamide derivatives of the activity of human 8-oxoguanine DNA glycosylase (OGG1) for several products of oxidatively induced DNA base lesions. ACS Chem. Biol. 2021, 16 (1), 45–51. 10.1021/acschembio.0c00877. 33331782 PMC9199349

[ref38] Tanner L.; Single A. B.; Bhongir R. K. V.; Heusel M.; Mohanty T.; Karlsson C. A. Q.; Pan L.; Clausson C. M.; Bergwik J.; Wang K.; Andersson C. K.; Oommen R. M.; Erjefält J. S.; Malmström J.; Wallner O.; Boldogh I.; Helleday T.; Kalderén C.; Egesten A. Small-molecule-mediated OGG1 inhibition attenuates pulmonary inflammation and lung fibrosis in a murine lung fibrosis model. Nat. Commun. 2023, 14 (1), 643 10.1038/s41467-023-36314-5. 36746968 PMC9902543

[ref39] Wilson D. L.; Kool E. T. Fluorescent probes of DNA repair. ACS Chem. Biol. 2018, 13 (7), 1721–1733. 10.1021/acschembio.7b00919. 29156135 PMC5976523

[ref40] Mabley J. G.; Pacher P.; Deb A.; Wallace R.; Elder R. H.; Szabo C. Potential role for 8-oxoguanine DNA glycosylase in regulating inflammation. Faseb J. 2005, 19 (2), 290–292. 10.1096/fj.04-2278fje. 15677345

[ref41] Bacsi A.; Aguilera-Aguirre L.; Szczesny B.; Radak Z.; Hazra T. K.; Sur S.; Ba X.; Boldogh I. Down-regulation of 8-oxoguanine DNA glycosylase 1 expression in the airway epithelium ameliorates allergic lung inflammation. DNA Repair 2013, 12 (1), 18–26. 10.1016/j.dnarep.2012.10.002. 23127499 PMC3678389

[ref42] Ba X.; Bacsi A.; Luo J.; Aguilera-Aguirre L.; Zeng X.; Radak Z.; Brasier A. R.; Boldogh I. 8-oxoguanine DNA glycosylase-1 augments proinflammatory gene expression by facilitating the recruitment of site-specific transcription factors. J. Immunol. 2014, 192 (5), 2384–2394. 10.4049/jimmunol.1302472. 24489103 PMC3943862

[ref43] Ba X.; Aguilera-Aguirre L.; Rashid Q. T.; Bacsi A.; Radak Z.; Sur S.; Hosoki K.; Hegde M. L.; Boldogh I. The role of 8-oxoguanine DNA glycosylase-1 in inflammation. Int. J. Mol. Sci. 2014, 15 (9), 16975–16997. 10.3390/ijms150916975. 25250913 PMC4200771

[ref44] Ba X.; Aguilera-Aguirre L.; Sur S.; Boldogh I. 8-Oxoguanine DNA glycosylase-1-driven DNA base excision repair: role in asthma pathogenesis. Curr. Opin. Allergy Clin. Immunol. 2015, 15 (1), 89–97. 10.1097/ACI.0000000000000135. 25486379 PMC4364697

[ref45] Boldogh I.; Pan L.; Vlahopoulos S.; Zheng X.; Wang K.; Hazra T. K.; Hegde M. L.; Bacsi A.; Radak Z.; Brasier A. R., OGG1 at the crossroads of inflammation and DNA base excision repair. DNA Damage, DNA Repair and Disease. Dizdaroglu M.; Lloyd R. S.. Royal Society of Chemistry, 2021, 75–103.

[ref46] Girard P. M.; D’Ham C.; Cadet J.; Boiteux S. Opposite base-dependent excision of 7,8-dihydro-8-oxoadenine by the Ogg1 protein of Saccharomyces cerevisiae. Carcinogenesis 1998, 19 (7), 1299–1305. 10.1093/carcin/19.7.1299. 9683192

[ref47] Zharkov D. O.; Rosenquist T. A.; Gerchman S. E.; Grollman A. P. Substrate specificity and reaction mechanism of murine 8-oxoguanine-DNA glycosylase. J. Biol. Chem. 2000, 275 (37), 28607–28617. 10.1074/jbc.M002441200. 10884383

[ref48] Fabbrizi M. R.; Nickson C. M.; Hughes J. R.; Robinson E. A.; Vaidya K.; Rubbi C. P.; Kacperek A.; Bryant H. E.; Helleday T.; Parsons J. L. Targeting OGG1 and PARG radiosensitises head and neck cancer cells to high-LET protons through complex DNA damage persistence. Cell Death Disease 2024, 15 (2), 150 10.1038/s41419-024-06541-9. 38368415 PMC10874437

[ref49] Roy L. M.; Jaruga P.; Wood T. G.; McCullough A. K.; Dizdaroglu M.; Lloyd R. S. Human polymorphic variants of the NEIL1 DNA glycosylase. J. Biol. Chem. 2007, 282 (21), 15790–15798. 10.1074/jbc.M610626200. 17389588

[ref50] Reddy P. T.; Jaruga P.; Kirkali G.; Tuna G.; Nelson B. C.; Dizdaroglu M. Identification and quantification of human DNA repair protein NEIL1 by liquid chromatography/isotope-dilution tandem mass spectrometry. J. Proteome Res. 2013, 12 (2), 1049–1061. 10.1021/pr301037t. 23268652

[ref51] Jaruga P.; Kirkali G.; Dizdaroglu M. Measurement of formamidopyrimidines in DNA. Free Radical Biol. Med. 2008, 45 (12), 1601–1609. 10.1016/j.freeradbiomed.2008.09.019. 18926902

[ref52] Dizdaroglu M.; Coskun E.; Jaruga P. Measurement of oxidatively induced DNA damage and its repair, by mass spectrometric techniques. Free Rad. Res. 2015, 49 (5), 525–548. 10.3109/10715762.2015.1014814. 25812590

[ref53] Jaruga P.; Coskun E.; Kimbrough K.; Jacob A.; Johnson W. E.; Dizdaroglu M. Biomarkers of oxidatively induced DNA damage in dreissenid mussels: A genotoxicity assessment tool for the Laurentian Great Lakes. Environ. Toxicol. 2017, 32 (9), 2144–2153. 10.1002/tox.22427. 28568507 PMC5669367

[ref54] Boiteux S.; Gajewski E.; Laval J.; Dizdaroglu M. Substrate-Specificity of the Escherichia-Coli Fpg Protein (Formamidopyrimidine DNA Glycosylase) - Excision of Purine Lesions in DNA Produced by Ionizing-Radiation or Photosensitization. Biochemistry 1992, 31 (1), 106–110. 10.1021/bi00116a016. 1731864

[ref55] Karakaya A.; Jaruga P.; Bohr V. A.; Grollman A. P.; Dizdaroglu M. Kinetics of excision of purine lesions from DNA by Escherichia coli Fpg protein. Nucleic Acids Res. 1997, 25 (3), 474–479. 10.1093/nar/25.3.474. 9016584 PMC146462

